# Evolutionary constraints permeate large metabolic networks

**DOI:** 10.1186/1471-2148-9-231

**Published:** 2009-09-11

**Authors:** Andreas Wagner

**Affiliations:** 1 University of Zurich, Dept. of Biochemistry, Bldg. Y27, Winterthurerstrasse 190 CH-8057 Zurich, Switzerland; 2 Department of Biology, University of New Mexico, Albuquerque, New Mexico, USA; 3 The Santa Fe Institute, Santa Fe New Mexico, USA; 4 The Swiss Institute of Bioinformatics, Quartier Sorge - Batiment Genopode, 1015 Lausanne, Switzerland

## Abstract

**Background:**

Metabolic networks show great evolutionary plasticity, because they can differ substantially even among closely related prokaryotes. Any one metabolic network can also effectively compensate for the blockage of individual reactions by rerouting metabolic flux through other pathways. These observations, together with the continual discovery of new microbial metabolic pathways and enzymes, raise the possibility that metabolic networks are only weakly constrained in changing their complement of enzymatic reactions.

**Results:**

To ask whether this is the case, I characterized pairwise and higher-order associations in the co-occurrence of genes encoding metabolic enzymes in more than 200 completely sequenced representatives of prokaryotic genera. The majority of reactions show constrained evolution. Specifically, genes encoding most reactions tend to co-occur with genes encoding other reaction(s). Constrained reaction pairs occur in small sets whose number is substantially greater than expected by chance alone. Most such sets are associated with single biochemical pathways. The respective genes are not always tightly linked, which renders horizontal co-transfer of constrained reaction sets an unlikely sole cause for these patterns of association.

**Conclusion:**

Even a limited number of available genomes suffices to show that metabolic network evolution is highly constrained by reaction combinations that are favored by natural selection. With increasing numbers of completely sequenced genomes, an evolutionary constraint-based approach may enable a detailed characterization of co-evolving metabolic modules.

## Background

Evolutionary history can constrain future evolution. It can constrain both the production and the preservation of phenotypic variation [1-6]. For instance, the acquisition of some traits may require the presence of other traits. To take a metabolic example, the biosynthesis of steroid hormones uses cholesterol as its starting point and prerequisite [7]. Cholesterol, in turn is a eukaryotic invention. Conversely, the loss of certain traits constrains future evolution, because it may be difficult to reverse, as exemplified by the independent and irreversible loss of planktonic feeding stages in multiple echinoderms [8].

The best-studied cases of constrained variation regard macroscopic and readily observable organismal traits [9]. However, if one wants to study genetic contributions to constrained variation, such traits are not ideal study objects. This is because hundreds to thousands of genes with often poorly understood interactions are typically involved in forming any macroscopic trait. Such incomplete characterization of genotypes, and of how exactly they produce phenotypes render genetic causes of constrained evolution difficult to understand for complex traits.

This problem suggests that more tractable genetic systems, where genotypic information is readily available, may be a useful starting point to learn more about the extent and pervasiveness of evolutionary constraints. Molecules such as proteins and RNA are the best-studied such candidate systems, but regulatory and metabolic networks are increasingly accessible with the available of genome-scale sequence and functional data [10-34]. Taken together, the phenotypic diversity of molecules and the networks they form is sufficiently rich to encapsulate the phenotypic diversity of organismal traits. Especially for metabolic networks, significant amounts of information about network genotypes and how they vary among species are available [35-37].

Studies of evolutionary constraints as applied to DNA, RNA, or protein sequences have a long history [38,39]. They show that most amino acid or nucleotide residues of these molecules cannot vary freely, and that variation in some residues is much more constrained than in others. Only a minority of residues may be under weak or no constraint, for example those that cause silent changes in lowly expressed proteins. We know less about evolutionary constraints for biological networks such as genome-scale metabolic networks, despite intriguing experimental observations that raise many questions about such constraints. Specifically, gene knockout experiments and computational work [13,14,16,22,26,40-44] show that in any one environment, many individual reactions of a metabolic network are expendable. Even reactions in the most central parts of metabolism, such as glycolysis or the citric acid cycle may be dispensable [14]. One reason lies in the distributed nature of metabolic systems, where several bypasses may exist around any blocked pathway. Does that mean that metabolic networks are unconstrained, or only weakly constrained in changing their complement of enzymatic reactions on evolutionary time scales?

I will here ask this and related questions with data from more than 200 prokaryotic genome-scale metabolic networks. Such networks are central to all life. They sustain it by producing metabolic energy and biosynthetic precursors. The metabolic network of typical free-living heterotrophic organisms comprises of the order of 10^3 ^different biochemical reactions [40,45,46]. These reactions are catalyzed by enzymes which are encoded by genes. Variation in the structure of such a network occurs through either mutational elimination of individual reactions (enzyme-coding genes), or through addition of one or more reactions, for which horizontal gene transfer is a major mechanism in prokaryotes. Information about which reactions are catalyzed in any one organism has been assembled into various databases [36,37,47] through a combination of manual curation and comparative genome analysis. For example, the KEGG database whose data I use here contains information about the complement of enzymes encoded in more than 600 completely sequenced prokaryotic genomes.

Can individual reactions in a metabolic network vary independently from other such reactions? If so, what fraction of reactions can vary independently? If co-variation among reactions occurs, does it affect pairs of enzymes or larger groupings? Only a few years ago, these and similar questions could not have been addressed, because the number of completely sequenced genomes required for at least a coarse metabolic annotation [36] was too small. With genome-scale information for hundreds of organisms, such analysis is now becoming tractable.

## Results

### High diversity of metabolic networks

The elementary unit of evolutionary change in metabolic networks is the individual chemical reaction catalyzed by an enzyme that is encoded by a metabolic gene. Except where mentioned otherwise, I here represent each such reaction on the level of the gene, as represented by orthologs of metabolic enzyme-coding genes in the Kyoto Encyclopedia of Genes and Genomes (KEGG) database (; [36]). This representation facilitates evolutionary analysis, because it is genes (and not reactions) that undergo mutations, and that are exchanged between organisms through horizontal gene transfer.

**Figure 1:**
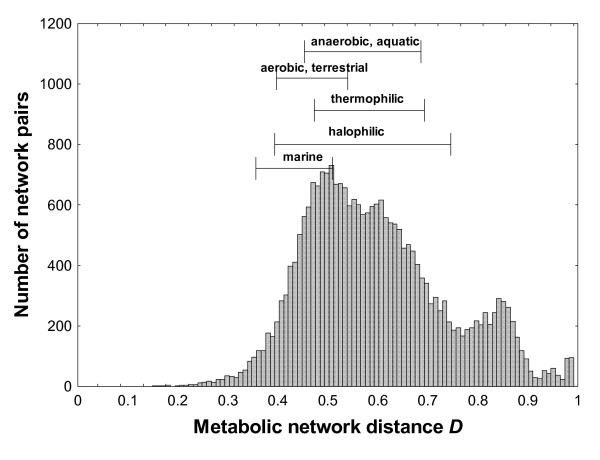
**Metabolic networks can show very different composition that depends on their evolutionary distance**. The figure shows a histogram of the fraction *D *of reactions (represented by KEGG orthologs; [36]) that occur in only one network of a pair of metabolic networks. The histogram is based on all networks that occur in 222 prokaryotes with completely sequenced genomes. Horizontal bars indicate mean (center of bar) and one standard deviation (length of bar) of *D *for organisms that live in the habitat-type indicated above each bar (see Methods for details).

Before studying constrained variation in individual reactions, it is useful to ask how diverse the composition of different metabolic networks really is. Previous work focused on different questions has assessed different aspects of this diversity [29,48-51], but genome-scale data about metabolic networks is accumulating so rapidly that continued assessments are useful. I used metabolic network data from 648 prokaryotic species in KEGG. To avoid biasing the analysis towards very closely related species, I focus for the rest of this contribution on one representative of each prokaryotic genus or 222 metabolic networks in total (median number of 1057 reactions per network). For each pair of these networks, I first determined how different their complement of chemical reactions was, by calculating the fraction *D *of reactions that occurs in only one but not both of the two networks. (*D *= 1 for networks that share no reactions.) Figure 1 shows this distribution of *D*. Its large mean of *D *= 0.68 suggests that two networks share on average only about one third of their reactions. Superimposed on the distribution of Figure 1 are data (horizontal bars) that indicate the mean (center of bar) and standard deviation (length of bar) of *D *for prokaryotic taxa that share a similar, broadly defined habitat. Metabolic networks are not much less diverse in these habitats than in the whole data set of metabolic networks. Even 13 different strains of *E. coli *show a mean *D *= 0.36 (s. dev. σ = 31; median *D *= 0.25).

### Many constrained reaction pairs

These observations suggest that metabolic networks are very diverse in their complement of metabolic reactions, even for organisms living in similar habitats. Together with the resilience of such networks to elimination of reactions [13,14,16,22,26,40-44], which indicate enormous plasticity in metabolic network organization in a given environment, they raise the question whether the evolution of such networks is perhaps only subject to weak constraints. I first addressed this question on the smallest level of evolutionary change, that of the individual reaction.

**Figure 2:**
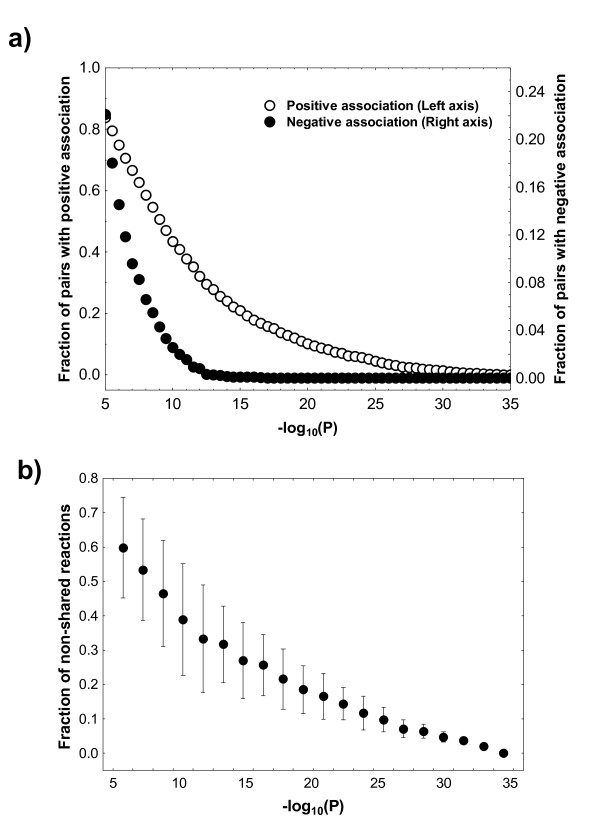
**Many reactions show constrained pairwise evolution**. **a) **The horizontal axis shows the negative decadic P-value of the statistical association of two reactions, as determined by an exact binomial test (see Methods). The left vertical axes shows the proportion of reactions that are positively associated, that is, that co-occur more often than expected by chance alone. The right vertical axis shows reactions with negative associations, reactions that occur less often together than expected by chance alone. Note the difference in scale for the two vertical axes. The Bonferroni-corrected value of P = 0.05 (0.05/(1.35 × 10^7^)) lies at -log_10_(P) = 8.43. **b) **The vertical axis shows the mean and standard deviation (length of bars) of the number of genomes that harbor only one but not both reactions of a positively associated reaction pair. Specifically, if two reactions are encoded by *n*_1 _and *n*_2 _genomes, and if *n*_12 _genomes encode both reactions, then the vertical axis shows the quantity 1-(*n*_12_/(*n*_1_+*n*_2_-*n*_12_)), averaged over all reaction pairs whose P-value lies in a given range (horizontal axis).

On this level, evolution would be unconstrained for any one reaction, if the occurrence of the reaction in a metabolic network can vary independently of other reactions. The most-straightforward way to assess this kind of constraint is to study statistical associations among all pairs of reactions. To this end, I applied an exact binomial test to the 1.35 × 10^7 ^possible pairs of the 5188 reactions found in the 222 networks I studied. This test determines whether two reactions occur jointly in these networks more often than expected by chance alone. Among several approaches to account for multiple testing [52,53], I here choose the (highly conservative) Bonferroni correction, focusing on reaction pairs with P-values below a Bonferroni-corrected P = 0.05, i.e., P = 0.05/(1.35 × 10^7^) = 3.7 × 10^-9^. Figure 2a shows the proportion of reactions that are associated with at least one other reaction according to this test, at a P-value exceeding the value shown on the horizontal axis. The figure shows that about half of reactions are associated with some other reaction at the Bonferroni-corrected P = 0.05. Individual reaction pairs can have P-values as high as10^-35^. It is important to note, however, that the association of two reactions, that is, their co-occurrence in the same genome, is rarely perfect. This is illustrated in Figure 2b, which shows for all associated reaction pairs, as a function of P-value the mean (± one standard deviation) fraction of genomes that encode only one but not the other reaction. For any constrained reaction pair, a value of 0.5 would mean that half of the examined genomes encode one but not both reactions. The figure shows that the fraction of genomes encoding only one reaction is greater than 20 percent except for the most highly constrained reaction pairs.

The same kind of test can also be used to ask whether there are pairs of reactions that tend to "avoid" each other, that is, whether a network that harbors one reaction tends not to harbor the other reaction. Such reaction pairs exist, but their numbers are much smaller. Specifically, fewer than five percent of reactions show such a negative association among genomes at the Bonferroni-corrected P = 0.05, and no reaction pair shows a P-value smaller than 10^-17 ^(Figure 2a). In sum, these associations suggest that a substantial fraction of reactions covary extensively with other reactions.

### Many constrained reaction sets

In any metabolic network, the production of important metabolites for biomass production requires the cooperation of multiple reactions. This observation calls for an extension of the above reaction-centered approach to larger units. Co-occurrence of reaction sets would suggest joint constrained evolution and joint requirement for key metabolic processes. A substantial technical problem to identifying such sets is the astronomical number of possible combinations (triples, quadruples, and so forth) of reactions, which renders an exhaustive evaluation infeasible. To circumvent this problem, I take the following graph-theoretic approach. I define a *reaction constraint graph *whose nodes are individual reactions. Two reactions are connected by an undirected edge in this graph if one of the reactions is associated with another reaction in the pairwise assay above, at a P-value that lies below a given threshold. A connected component in this graph is defined as a set of reactions that shows pairwise associations among set members, but where no reaction is associated with other reactions outside the set. (I eliminate isolated reactions, that is, components of size one from this graph.) Such connected components can be thought of as sets of constrained or co-occurring reactions, and their identification is the target of this part of my analysis. The size and number of components of the reaction constraint graph may vary, depending on which P-value threshold is used to define the graph. At a high threshold (low required statistical significance), the graph may consist only of one or few large components that comprise most reactions, whereas at lower thresholds, the graph may fragment into multiple components of decreasing size that indicate increasingly strongly associated sets of reactions. To have a frame of reference, I compared the structure of this graph at any given threshold to that of a randomized graph. This randomized graph was generated from the original reaction association graph through swapping of edge pairs (see Methods), which leaves the number of edges, and the number of edges per node constant, but randomizes the graph in other respects. More specifically, I generated 20 such randomized graphs and characterized the component size distributions of each of these graphs.

**Figure 3:**
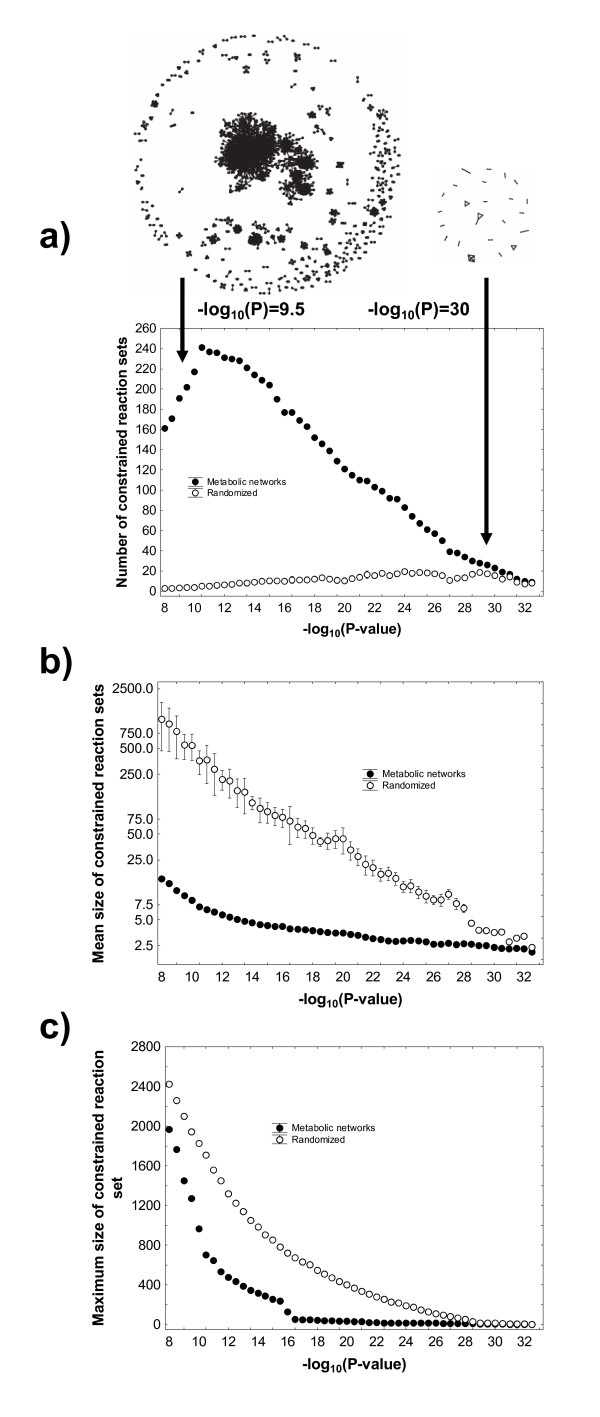
**Reaction constraint graphs show many more and smaller constrained reaction sets than randomized graphs**. The plots show **a) **the number of components, **b) **the mean size of components, and **c) **the maximum component size (vertical axes), as a function of the negative decadic logarithm of the significance threshold P (horizontal axis), for reaction constraint graphs (closed circles) and randomized versions of these graphs (open circles). Randomization was carried out with an edge swapping algorithm [102] (see Methods) that preserves the graph's degree distribution. All data for random reaction graphs are based on 20 randomized graphs for each significance threshold. Error bars for randomized graphs indicate one standard deviation. Where invisible, standard deviations are too small to be shown. The two graphs drawn above panel a) show the structure of the reaction constraint graph at two significance thresholds, -log_10_(P) = 9.5 (left) and -log_10_(P) = 30 (right).

**Figure 4:**
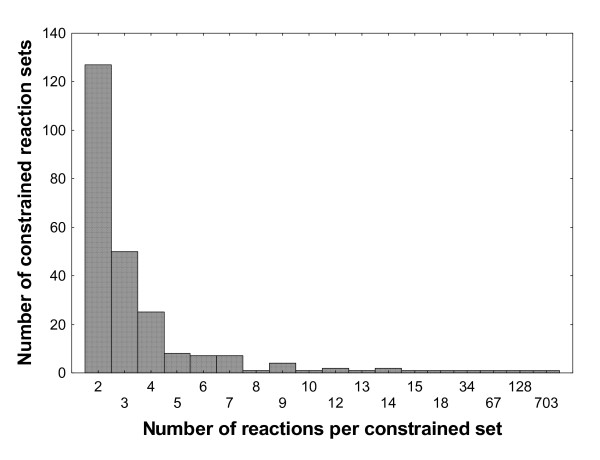
**Most highly constrained reaction sets are small**. The figure shows a histogram of the number of reactions per constrained reaction set (connected component) for the reaction constraint graph at -log_10_(P) = 10.5, where the total number of components is close to a maximum (see Figure 3a).

Figure 3a shows that at even low P-values, the reaction constraint graph fragments into many constrained reaction sets. Specifically, even at a P-value close to the Bonferroni-corrected P = 0.05, this graph has 202 such sets, with a mean number of 9.6 (standard deviation 89.3; Figure 3b) reactions per set, and a wide variation from 120 sets with only 2 reactions to one large component with 1271 reactions (Figure 3c). Over most of the P-value range explored, the number of constrained reaction sets is orders of magnitudes larger than the corresponding number in randomized graphs. For example, at the Bonferroni-corrected P = 0.05, randomized constraint graphs have on average 50-fold fewer components (mean 3.85 components; standard deviation σ = 1.03). The number of constrained reaction sets declines in randomized graphs, but not in the actual reaction constraint graph, where it increases with decreasing P-value to a peak of 241 such sets (at P = 10^-10.5^) and then declines steadily. Figure 4 shows the distribution of the number of constrained reaction sets for the P-value where the number of such sets is the largest (P = 10^-10.5^). At other P-values, this distribution is qualitatively similar. Clearly, the overwhelming majority of reactions are associated in small sets of two, three, or four reactions, and there are only few larger reaction sets. The mean and maximal component sizes are generally smaller for the actual randomized reaction constraint graph than for randomized graphs (Figure 3b and 3c). The mean number of reactions per set, as well as the size of the largest sets decline with increasing P for both the actual and the randomized graphs (Figures 3b and 3c).

### Constrained reaction sets and metabolic pathways

In sum, sets of evolutionary constrained reactions are typically small, much smaller than would be expected if reaction associations were distributed randomly across metabolic networks. These observations raise the question whether the constrained sets of reactions are largely congruent with traditional classifications of metabolic pathways. To address this question, I took advantage of the biochemical pathway classification of reactions in KEGG [36]. For any constrained reaction set as defined above, I determined the fraction of all reaction pairs that are assigned to the same metabolic pathway. For those reaction sets at the Bonferroni-corrected P = 0.05 where reactions can be assigned to individual pathways, all reaction pairs within a set can be assigned to the same pathway for almost 90% (124/139) of sets. More than half of the remaining few (15) reaction sets have between four and 1271 reactions, and are thus biased towards larger reaction sets. For more tightly associated reaction sets (very small P-values), this bias towards constrained reactions in the same pathway increases. For example, at P = 10^-15 ^and P = 10^-20^, respectively 92% (127/138) and 96.6% (85/88) constrained reaction sets belong in one pathway. In sum, individual reactions for most constrained reaction sets can be allocated to the same pathway.

### Specific examples: Top 15 reactions

Table 1 shows the 15 most highly constrained reaction sets, that is, those sets with the smallest P-value. The negative decadic logarithm of this P-value is indicated in column 1 from the left. All of the reaction sets in the Table have P < 10^-16^. Column 2 shows the number of reactions in each set. In keeping with the skewed distribution of reaction set sizes (Figure 4), most sets have size two, and only few sets are larger. Column 3 shows either the metabolic pathway a reaction set belongs to, or the individual reactions where this pathway annotation is unknown or highly ambiguous. Most constrained reaction sets have a clear pathway affiliation, and only few sets involve proteins of unknown or poorly characterized function. The sets also occur in a great diversity of pathways, including amino acid metabolism, carbohydrate biosynthesis, and butanoate metabolism. Noteworthy is that several cofactor biosyntheses appear among the most highly constrained reaction sets They include the synthesis pathways of cobalamin (vitamin B_12_); biotin (vitamin H or B_7_), a cofactor in fatty acid and leucine biosynthesis, and pyrroloquinoline quinone (PQQ), a redox cofactor. Such coenzymes have a complex molecular structure and complex biosynthetic pathways that are not situated at the center of an organism's metabolic network, but rather at the periphery. For these reasons they are often less reticulate than the most central parts of metabolism, with fewer alternative routes between metabolic intermediates. This may be the very reason why they are highly constrained: Absence of an individual reaction in such peripheral, complex pathways may not be as easily compensated through reactions in alternative pathways [17]. The production of any one complex cofactor may then require that specific sets of reactions are present.

**Table 1:** The 15 most highly constrained sets of metabolic reactions.

**P_min_**	**Reactions**	**Pathways/Function**	**KEGG Identifier**
22.73	3	Histidine degradation	K01468 K01712 K01745
21.93	2	Unknown/methyltransferase	K06346 K06960
21.56	2	Transmembrane sensor/Copper resistance	K07156 K07245
21.45	2	Glycosyltransferase/Glucan biosynthesis	K03669 K03670
21.22	2	Glutamate metabolism	K00620 K00642
18.04	3	Pyrroloquinoline quinone (PQQ) biosynthesis	K06139 K06136 K06138
17.58	4	Cobalamin biosynthesis	K02232 K02227 K02233 K02231
17.51	2	Glutamate-ammonia-ligase adenylyltransferase/uridylyltransferase	K00982 K00990
17.17	2	Biotin biosynthesis	K00652 K01935
17.06	2	Acetyl CoA, fatty acid and amino acid metabolism	K00022 K01692
17.01	4	Inositol phosphate catabolism	K03335 K03336 K03337 K03338
16.84	2	Starch and glycogen biosynthesis	K00700 K00975
16.77	2	4-hydroxy 3-oxovalerate aldolase/acetaldehyde dehydrogenase	K01666 K04073
16.69	2	Butanoate Metabolism	K00023 K03821

Column 1 from the left shows the negative decadic logarithm of the lowest P-value for a reaction pair within a constrained reaction set. That is, for constrained reaction sets comprising more than two reactions, all reaction pairs have a P-value lower than that indicated in this column by the Table. Column 2 shows the number of reactions in each set. Column 3 shows, for reaction sets with a known pathway annotation, the respective biochemical pathway [36], or, where the pathway is not known or ambiguous, the functions of the enzymes, separated by a slash. Column 4 shows the unique KEGG identifiers [36] for the respective enzyme-coding genes.

The same causes may also explain the conspicuous absence of reactions in the most central parts of metabolism in the set of Table 1, despite the importance of such reactions in life. Central carbon metabolism is highly reticulate, with many alternative metabolic routes for missing reactions [17], which may lead to fewer highly constrained reaction sets.

### Specific examples: Histidine degradation

**Figure 5:**
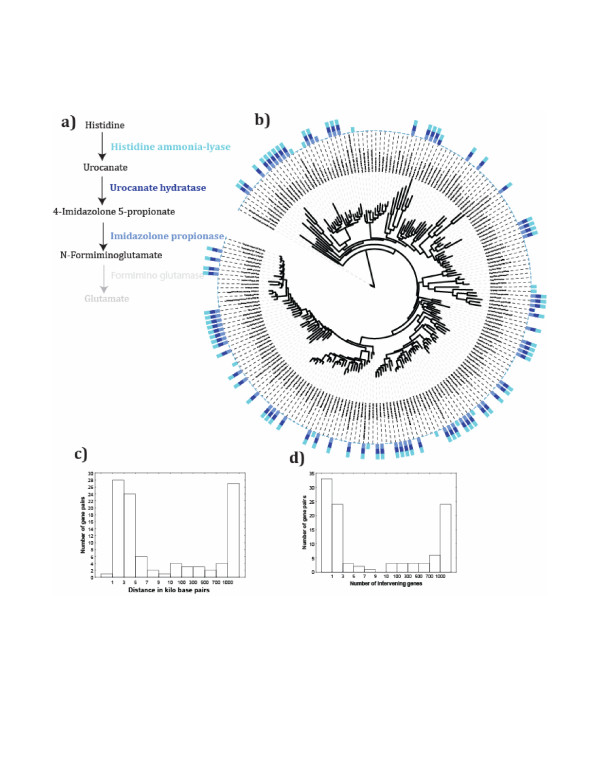
**Three highly constrained reactions (P < 10^-20^) in the histidine utilization pathway**. **a) **shows the three first reactions of the histidine utilization pathway, color-coded to help visualize their occurrence in **b)**, which displays a 16S rDNA-based maximum-likelihood phylogenetic tree, visualized by ITOL [101], of the bacterial species analyzed here. Bars along the circumference of the tree indicate whether a specific reaction (as indicated by the bar's color) is encoded by a genome or not. Bars containing two or more colors indicate that the respective reactions are encoded in two or more genomes. Note that most bars contain all three colors, indicating that the respective genomes encode all three reactions. **c) **shows the distance in kilobase pairs and **d) **the number of genes intervening between genes encoding histidine ammonia lyase and imidazolone propionase in the studied genomes. The bimodality of the distributions in c) and d) is similar for the other reaction pairs (not shown), with a bias towards tightly linked genes. The fourth reaction (formiminoglutamase) shown in a) is not part of a highly constrained reaction set significant at P < 10^-20^. However, it is associated with the remainder of the pathway. For example, it is associated with the imidazolonepropionase reaction preceding it at P < 10^-8^. Whereas 84 genomes encode imidazolonepropionase, only 38 of them encode a known ortholog of formiminoglutamase, which is responsible for the weaker association. (37 of these 38 genomes also encode the imidazolonepropionase reaction.)

I will next focus on those reaction sets in Table 1 that involve more than just two reactions, and where the respective pathway is well-characterized. The first set (row one of Table 1) contains the first three reactions in the degradation of histidine to glutamate, a pathway that is responsible for the utilization of histidine, and that ultimately feeds into the citric acid cycle. (Figure 5a). Each of the reactions occurs in between 84 and 95 of the studied genomes. Together they form a highly significant reaction set (P < 10^-23 ^for each of the three possible reaction pairs), whose congruence in genomic association is nearly perfect. Figure 5b illustrates this association with a 16S rDNA-based phylogenetic tree of the analyzed species and, along the circumference of this tree, color-coded bars that indicate the presence or absence of each reaction. For example, the topmost two reactions of Figure 5a occur in 84 and 85 genomes, respectively, and 83 of these genomes encode both reactions. The tree also shows that most genomes encode either all three reactions or none of them. Moreover, the species encoding these reactions are scattered throughout the tree, which reflects a broad range of both bacterial and archaeal taxa. This suggests that the association among these reactions is no artefact of their vertical co-inheritance among lineages represented on the tree. This is confirmed by a matched pair test [54,55], which can take the structure of a phylogenetic tree in association testing into account (association significant at P < 2.3 × 10^-13^, for each of the three reaction pairs).

I next asked whether constrained gene pairs in this set are always tightly linked, which might indicate that they are always transferred jointly. Figure 5c shows a histogram of the distance between the genes encoding the first and third reaction in the pathway of Figure 5a, for all genomes that encode both of these reactions. Figure 5d shows an analogous histogram for the distance in terms of the number of intervening genes. Perhaps the most striking feature of this distribution is its bimodality. That is, a substantial fraction of gene pairs seems to be tightly linked, with a distance of fewer than 10 kilo base pairs and fewer than 10 intervening genes, but an equally substantial fraction is loosely linked or unlinked, with more than 100 kbp and hundreds of genes in between them. The tightly linked gene pairs likely reflect the well-known organization of histidine utilization genes into one or two linked operons, which has been observed for some organisms [56-58]. The existence of many unlinked gene pairs suggests that not all of the covariation of these genes can be explained through constrained variation. Bimodial distributions (not shown) are also observed for the other two reaction pairs analyzed in Figure 5a.

### Specific examples: Cobalamin, PQQ, and inositol metabolism

**Figure 6:**
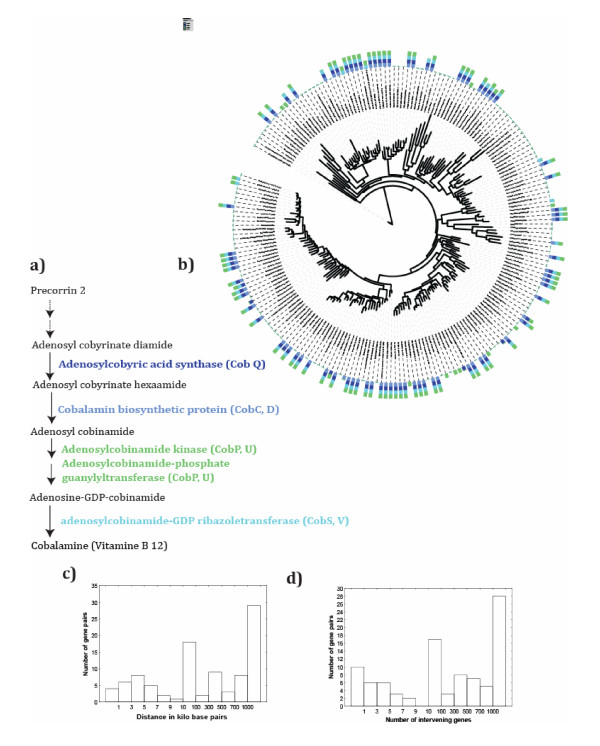
**Four highly constrained reactions (P < 10^-20^) in cobalamin biosynthesis**. **a) **shows the four last reactions of the cobalamin biosynthesis pathway, color-coded to help visualize their occurrence in **b)**, which displays a 16S rDNA-based maximum-likelihood phylogenetic tree of the bacterial species analyzed here. Bars along the circumference of the tree indicate whether a specific reaction (as indicated by the bar's color) occurs in a genome or not. Bars containing two or more colors indicate that two or more reactions occur in a given species. Note that most bars contain all four colors, indicating that the respective genomes encode all three reactions. Gene symbols 'Cob*' in a) reflect names of genes known to catalyze these reactions in aerobes [36,59]. **c) **shows the distance in kilobase pairs, and **d) **the number of genes intervening between the second and fourth reaction from a) in the studied genomes. The bimodality of this distribution is similar for the other reaction pairs (not shown).

A second example (row 7 in Table 1) concerns cobalamin (vitamin B_12_), one of the most complex biogenic small molecules. Its biosynthesis is restricted to prokaryotes [59]. The last four reactions of this biosynthesis include the assembly of the major parts of the molecule into the final molecule [59]. These reactions form a highly significant association cluster (P < 10^-17 ^for each pair). The genes encoding these four reactions occur in between 92 and 98 of the examined genomes, and every pair of genes co-occurs in at least 82 genomes. Figure 6b shows that most taxa have either all or none of these genes, which are broadly distributed across the phylogeny, and not restricted to specific clades. (All pairwise associations are significant in a matched pair test at P < 9.1 × 10^-13^). As for histidine biosynthesis, the distance distributions of genes encoding cobalamin biosynthesis genes are bimodal, as shown in Figures 6c and 6d for the second and fourth reaction. Other reaction pairs also show bimodality in the distance distribution of their encoding genes (not shown). Individual reactions can but do not always co-occur in operons [59].

The remaining most highly constrained reaction sets of size greater than two occur in PQQ synthesis (3 reactions in 23-25 genomes; P < 10^-18^), whose reactions are poorly characterized, and in the catabolism of myo-inositol (4 reactions in 26-30 genomes; P < 10^-17^). As opposed to eukaryotes, where inositol derivatives have signaling roles, in prokaryotes they serve structural roles as membrane anchors of proteins and glycolipods, and they can aid the infectivity of pathogens [60]. Myo-inositol can also serve as sole carbon source for several microorganisms [61]. The reactions in question catalyze four consecutive steps in a pathway that converts myo-inositol into acetyl-CoA or glyceraldehyde-3-phosphate (KEGG pathway identifier: ko00562). In these last two examples, too few genomes contain the reactions to carry out a meaningful statistical analysis of the genomic distance distribution, but I note that also here, some of the genes encoding individual enzyme pairs are not closely linked (not shown).

**Figure 7:**
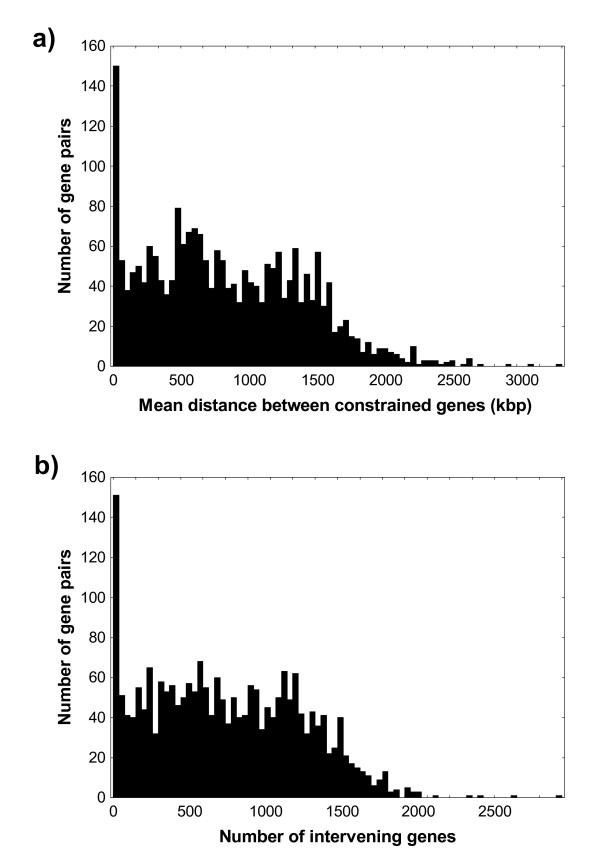
**The average distance between positively associated reactions is not necessarily small**. For all reaction pairs highly associated at P < 10^-20^, the panels show histograms of the mean distance in a) kilo base pairs, and b) number of genes between orthologs encoding the reactions in a pair.

This pattern, where constrained enzyme-coding gene pairs are not necessarily tightly linked, holds not only for the examples I just discussed. It holds much more generally, even for the most highly constrained pairs. Figure 7 shows the distribution of the mean distance between genes encoding constrained reaction pairs, either in kilo base pairs (Figure 7a) or in the number of intervening genes (Figure 7b) for pairs significant at P < 10^-20^. Although the distribution displays a distinct peak at short distances, it also makes clear that most genes are hundreds to thousands of kilo base pairs apart and are separated by hundreds of intervening genes. Such widely separated genes are likely to be transferred individually, not jointly. Thus, variational constraints may not be solely responsible for the constrained evolution of metabolic reactions.

### Specific examples: reactions with negative associations

**Figure 8:**
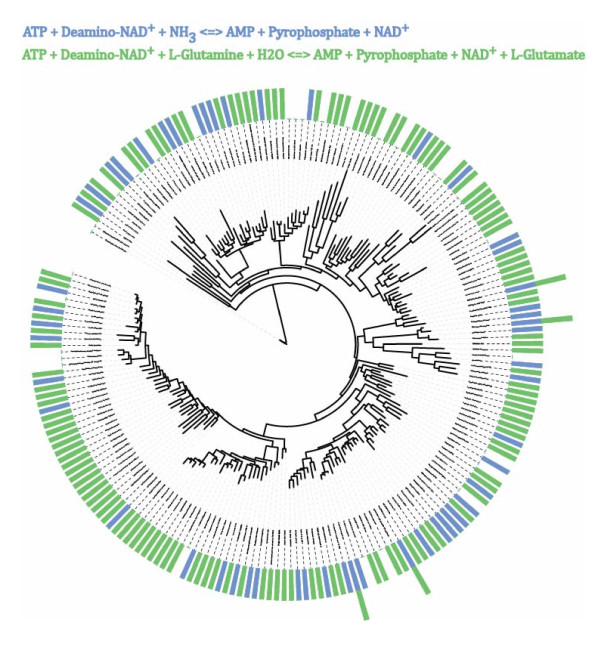
**Two NAD^+ ^synthase genes that "avoid" each other**. KEGG orthologs K01916 and K01950 are thought to encode NAD+ synthases that use ammonium (blue reaction) and glutamate (green reaction) as amide donors, respectively. The tree shown is a 16S rDNA-based maximum-likelihood phylogenetic tree of the bacterial species analyzed here. Bars along the circumference of the tree indicate whether a specific reaction (as indicated by the bar's color) occurs in a genome or not. Bars containing two colors indicate that both orthologs occur in a given species. These orthologs are highly negatively associated, as illustrated by their almost exclusively complementary distribution in the analyzed genomes.

A final class of examples comes from the (small) set of negative pairwise associations, where the occurrence of one reaction in a genome implies that the other reaction is absent. One might think that such associations might reflect alternative metabolic routes, where one route might exclude the presence of the other route in an organism, but this is not so. Earlier steps of cobalamin biosynthesis than those shown in Figure 6 provide an example. Specifically, the biosynthesis of adenosyl cobyrinate from precorrin 2 occurs according to two different routes, one that requires oxygen and another that does not [59]. However, sets of reactions in this and other alternate pathways are not generally negatively associated (results not shown). Instead, the strongest negative associations involve different individual enzymes that can carry out the same or similar reactions, and that show non-overlapping distributions among genomes. One example concerns two similar forms of the final reaction in the synthesis of the co-factor nicotinamide adenine dinucleotide (NAD), which use different amido group donors (Figure 8). Orthologs of the genes encoding these reactions occur in most examined genomes, but in an almost non-overlapping pattern (P < 10^-12^): Only four genomes contain orthologs for both reactions (Figure 8). Similar patterns of negative association occur for other enzymes, including a FAD (flavine adenine dinucletide)-dependent and FAD-independent thymidilate synthase (P < 10^-13^, [62,63]), an enzyme involved in the synthesis of the DNA building block dTMP, as well as two homoserine kinases (P < 10^-12^) and two prephenate dehydrogenases (P < 10^-11^). There are also several strong negative associations of unknown biological significance, such as that of the DNA mismatch repair protein MutS2 (KEGG ortholog identifier K02339) and the DNA polymerase holoenzyme subunit χ (K07456), which occur in 171 genomes but in only one of them jointly (P < 10^-15^).

## Discussion

Both experimental and computational work shows that metabolic networks vary greatly in their organization. For example, earlier work on the organization of the citric acid cycle in 19 completely sequenced microbes showed that almost every organism encodes a different subset of the cycle's reactions [50]. Given the centrality of this cycle in energy metabolism, this variability is especially remarkable. The genome-scale analysis of multiple metabolic networks from Figure 1 highlights such variability. It shows that metabolic networks are highly diverse in their reaction content. In addition, metabolic networks can be quite resilient to elimination of individual reactions in any given environment [13,14,16,22,26,40-44], partly because the blocked reactions can readily be bypassed through alternative metabolic routes. Furthermore, new metabolic pathways and reactions continue to be discovered [63,64]. Taken together, these observations raise the possibility that there are so many different ways of organizing the flow of matter through a metabolic network -- many of which still unknown -- that individual reactions may only be weakly constrained in their evolution. In this view, the mutational elimination of any one reaction might be readily compensated by an alternative metabolic route that is either already present in the genome or can be readily transferred into the genome through horizontal transfer.

In contrast to this scenario, the merely 222 genomes studied here suffice to show that the majority of reactions are indeed highly constrained in their evolution. This is indicated by their statistically significant co-occurrence with other reactions in constrained reaction pairs. The genes in such a pair are not always tightly linked, which renders horizontal co-transfer of constrained reaction sets an unlikely sole cause for these patterns of association. Constrained reaction pairs can be grouped into small sets whose number is substantially greater than would be expected if the same number of associations occurred among randomly chosen reaction pairs in a metabolic network. The reactions in a set typically belong to the same biochemical pathway(s). Despite the distributed nature of metabolic networks, where perturbations in one part of a large network can be compensated through changes in other, superficially remote parts, clearly identifiable constrained reaction sets exist, and are usually highly localized. These observations are consistent with earlier observations of modular structures in metabolic (and other) networks [29,51,65-73]. For example [70] focused on the identification of conserved modules in metabolic pathways, and showed that many such modules exist, have a skewed size distribution, and may be hierarchically organized.

In the bioinformatics community, questions regarding the modular organization of biological networks have attracted significant attention, unlike the topic of evolutionary constraints. In contrast, whether evolutionary constraints exist, and how pervasive they are has been an important and controversial topic in evolutionary biology. Standard textbooks [1] would discuss these questions extensively. This paper is complementary to earlier work from the bioinformatics community [29,51,65-73], not only in approach, but also by focusing on the concept of evolutionary constraints. It points to the fertile ground that data on molecular networks can provide for the analysis of such constraints.

In the evolutionary biology literature, constraints are hardly ever absolute. Classical examples include the tetrapod limb, which has mostly five digits, although ichthyosaurs had more; the lower jaw of frogs, which generally lacks teeth, except for the genus Gastrotheca [1]. Constraints are thus best viewed as statistical biases in the occurrence or co-occurrence of traits (here: chemical reactions). For metabolic networks, the reason behind the lack of absolute constraints are easily explained: Matter can flow along many alternative routes through a metabolic network. A reaction in a constrained reaction set whose presence is be essential in one metabolic network, may be dispensable in another, because its role can be assumed by other reactions. Statistical analyses like mine help avoid the opposite extreme of assuming maximal flexibility, because they show that network reactions show constrained evolution.

The tightly constrained evolution of many reaction sets raises a question about the causes of these constraints. Specifically, is it caused merely by natural selection favoring certain reaction combinations, or do metabolic reactions also co-vary in their transfer from bacterium to bacterium? An important source of variation in metabolic and other networks is horizontal gene transfer [21,74-82]. In such transfer, immediately adjacent genes are more likely to be transferred together in any one transfer event than more distantly related genes. For example, a study on horizontal transfer among five different *E. coli *species showed that the majority of transfer events involved fewer than 15 kilo base pairs of DNA [83]. Tight linkage of genes thus introduces a constraint, in addition to any constraint imposed by selection, because linked genes are not transferred independently of one another. Selection may of course itself be the ultimate cause of such tight linkage, because tight linkage may allow beneficial coregulation or co-transfer of genes [84-88]. In the latter case, selection's preference of certain gene combinations may have caused variation in these gene combinations itself to become constrained. In other words, if certain reaction combinations are favorable, then the genes involved in them might become tightly linked over time.

I asked how important gene linkage is in constraining reaction evolution by examining the most highly constrained reaction pairs and how closely together their encoding genes are linked in a genome. Not surprisingly, the results show clear evidence that some such linkage occurs. However, there are also many cases where linkage is not likely to be solely responsible for constrained variation. The reason is that covarying metabolic genes are often unlinked in many genomes in which they occur. This observation is in line with previous work [89], which suggested that gene clusters and operons are highly dynamic on an evolutionary time scale. They form and disintegrate readily, and their constituent genes are sometimes tightly linked, sometimes scattered throughout the genome [84,86,89,90].

Horizontal gene transfer is perhaps the most rapidly acting form of change in prokaryotic metabolic networks, because it can introduce multiple new genes into a genome on short evolutionary time scales. However, metabolic networks can change also without such horizontal transfer, albeit on longer time scales. Aside from the mutational loss of reactions (which may be slow for reactions in a highly constrained reaction set), new enzymes can be created through gene duplication and subsequent sequence divergence, as well as through recombination and shuffling of domains and exons [91-94]. Such processes are the source of new enzymatic reactions that can then be "shared" among organisms through horizontal transfer. Their successful transfer will depend on whether reactions of in the same constrained reaction set are co-transferred, or are already present in the new host.

Current limitations of any statistical approach to analyze constrained evolution of metabolic networks include the limited number of available genomes. With hundreds of genomes at hand, such statistical characterization is beginning to be meaningful, but it is unlikely to resolve the fine-structure of such associations, for example by distinguishing more conserved from less conserved pathways with any confidence. The observation that some 20 percent of reactions appear to be unconstrained (Figure 2a) may be explicable through this limitation. Some reactions occur only in few of the 222 genomes I studied, and to preserve a meaningful statistical analysis, I excluded reaction pairs whose reactions occurred in fewer than five genomes. The further the number of genomes that contain each reaction in a pair deviates from this lower limit, the more readily a statistical test can reveal constraints. Thus, the number of apparently unconstrained reaction pairs will undoubtedly decrease as the number of completely sequenced genomes increases. It may well decrease to zero. Similarly, metabolic databases contain annotation errors. Their numbers are unknown, and their presence is a source of noise for such statistical analyses. Their incidence will undoubtedly decrease, as more genomes become sequenced and analyzed comparatively.

A second limitation comes from a particular class of potential network misannotation. A reaction can be catalyzed by an enzyme that is unrelated in sequence to any other known enzyme catalyzing this reaction. If so, then identification of metabolic network composition based on genome-sequence alone would miss the reaction. This is an example of convergent or parallel protein evolution, and has also been called orthologous gene replacement. For example, 5 out of 14 reactions in the citric acid cycle were reported to be subject to non-orthologous displacement [50]. (The respective genes are now all represented in KEGG). Unfortunately, manual curation of metabolic reactions becomes impractical in surveys of many metabolic networks. The problem is of unknown magnitude. It also will be alleviated only with time, as more unrelated enzymes encoding the same reactions are discovered.

Thirdly, the occurrence of particular pathways in data such as that of Table 1 depends on pathway annotation, and in particular on pathway size. Complex pathways with many reactions are more likely to harbor constrained reaction sets than small pathways with few reactions, merely by virtue of their larger size. In addition, different databases may contain somewhat different pathway annotations. Because of the dangers of overinterpreting constraints in particular pathways, I thus here focus only on more generic features, namely whether the reactions in a constrained set occur in the same pathway.

A final limitation is that the constrained reaction sets I analyze have evolved in the context of a phylogeny, but that statistical analysis neglects this evolutionary history. Some phylogenetic methods have been developed to address correlations caused by shared evolutionary history for phenotypic and sequence data [95]. Aside from their various limitations [95,96], such methods would have limited applicability to the microbial genomes that I study. First, most such methods require either an implicit or explicit model of character (here: reaction) evolution. Multiple such models exist for sequence data, because enormous amounts of such data exist that can inform these models. In contrast, such models do not yet exist for metabolic network evolution. Second, and more importantly, extensive horizontal transfer -- in particular of metabolic genes [75,78,83] -- obscures evolutionary history, which can differ greatly among reactions. If, for example, even genomes as closely related as those of different *Escherichia coli *strains differ in more than a megabasepair of sequence [83] and some 25% of metabolic reactions, then existing methods are surely inadequate for more distantly related species. Visual inspection and manual analysis is feasible for a small number of examples like those I study (Figures 5, 6, 8), but not for large numbers of constrained reaction sets. We sorely need new methods to incorporate evolutionary history and horizontal gene transfer into the analysis of genome-scale metabolic data sets.

## Conclusion

Despite the great apparent flexibility of metabolic networks suggested by gene knockout studies, and despite considerable network differences among closely related species, most individual reactions are not free to vary independently of other reactions. The discrepancy between these two observations can be resolved if one considers that the environment plays a crucial role in determining the effect of changing a network's reactions. Environmental variation is difficult to account for comprehensively in gene knockout studies, and its extent is often unknown for different species.

## Methods

16S rDNA data and phylogenetic analysis. All publicly available complete prokaryotic genome sequences were obtained in December 2008 from the National Center for Biotechnology Information (NCBI; ). One 16S rDNA sequence was extracted from the genbank file for each genome. 16S rDNA sequences were aligned using the NAST (Nearest Alignment Space Termination) algorithm, as implemented in a web server specifically designed to align 16S rDNA sequences (; [97]). Pairwise 16S rDNA nucleotide divergence was calculated from this multiple alignment as the fraction of (non-gap) characters that differ between two sequences. A prokaryotic 16S rDNA maximum-likelihood tree was constructed using the package phyml [98], with the Hasegawa-Kishino-Yano [99] substitution model, where the transition-transversion ratio and the proportion of variable sites were estimated from the data. To accommodate variable substitution rates among sites, I allowed for four different substitution rates and estimated the parameter of the gamma distribution determining the rate variation from the data. A tree generated by neighbor joining [100] was used as the starting tree to be refined by the maximum likelihood algorithm. ITOL [101] was used for tree visualization.

### Environmental data

NCBI provides a broad classification of habitat types for completely sequenced prokaryotes . From this classification, I identified 10 anaerobic aquatic species, 17 aerobic terrestrial species, 24 thermophilic species, and 8 moderate halophilic species for which complete genome sequences and metabolic network information were available. In addition, I identified seven completely sequenced marine prokaryotic species from the Marine Microbiology Initiative  with metabolic network information in KEGG. The horizontal bars in Figure 1 are based on these sets of species.

### Exact binomial test

If two reactions *R*_1 _and *R*_2 _occur in *n*_1 _and *n*_2 _networks, such that *n*_1 _≥ *n*_2_, and if *R*_2 _occurs independently from *R*_1_, then the number of metabolic networks harboring both *R*_1 _and *R*_2 _can be modeled as a binomially distributed random variable *X *with parameters *n*_1 _and *p*, where *p *is simply the total fraction of networks that harbor *R*_2_. To assess whether *R*_2 _co-occurs with *R*_1 _to a significantly greater extent than expected by chance alone, one can determine the probability P(*X *≥ *n*_12_), where *n*_12 _is the observed number of networks that harbor both *R*_1 _and *R*_2_. If P(*X *≥ *n*_12_) is smaller than a pre-determined significance threshold (e.g., P = 0.05) then the association of the two reactions is deemed significant. If *n*_1 _≤ *n*_2_, then the test is carried out with reversed roles of the two reactions *R*_1 _and *R*_2_. This test essentially asks if the less frequent of two reactions is significantly associated with the more frequent one. An exactly analogous test can be carried out to determine whether two reactions co-occur significantly less often than expected by chance alone. Specifically, values of 1- P(*X *≥ *n*_12_) that are smaller than a predetermined significance threshold would indicate reaction pairs with a tendency to *not *occur in the same metabolic network. In applications of these tests, I restricted myself to reaction pairs *R*_1 _and *R*_2 _where genes encoding these reactions occur in at least 5 genomes.

### Reaction constraint graph analysis

A reaction constraint graph is a graph whose nodes are reactions, and where two reactions are connected by an edge if the statistical significance of their pairwise association falls below a given significance threshold. To randomize such a graph, I used an edge swapping algorithm [102] that preserves each node's number of neighbors and the node's degree distribution. This algorithm first chooses two edges *e*_1 _and *e*_2 _at random that do not share any nodes. It then reconnects *e*_1 _such that its source node becomes linked to the target node of *e*_2_, and *e*_2 _such that its source node becomes linked to the target node of *e*_1_. I iterate this algorithm 2*E *times, where *E *is the total number of edges in the graph, to yield one randomized reaction graph. All results for random reaction graphs reported here are based on 20 randomized graphs for each significance threshold. To evaluate whether members of a constrained reaction set can be assigned to the same pathway, I first determined for each pair of members of this set whether they share at least one KEGG pathway annotation. I then calculated the fraction of pairs in the set for which this was the case.

### Distance of orthologs encoding associated reactions

For any two gene pairs that show a statistically significant association, I identified the names of all known orthologs of genes encoding these reactions from the KEGG "ko" file (available at ;[36]). I then searched for the respective genes and their genomic position in the annotated genbank genome sequence files available at . In those cases where one genome contains more than one ortholog encoding the same reaction, I calculated pairwise distances for each of these orthologs separately, and include these distances in the distributions reported here.
